# *Notes from the Field:* Targeted Biomonitoring for GenX and Other Per- and Polyfluoroalkyl Substances Following Detection of Drinking Water Contamination — North Carolina, 2018

**DOI:** 10.15585/mmwr.mm6829a4

**Published:** 2019-07-26

**Authors:** Jamie R. Pritchett, Jessica L. Rinsky, Beth Dittman, Ariel Christensen, Rick Langley, Zack Moore, Aaron T. Fleischauer, Kate Koehler, Antonia M. Calafat, Rachel Rogers, Laconial Esters, Rodney Jenkins, Faye Collins, Debra Conner, Patrick Breysse

**Affiliations:** ^1^Division of Public Health, North Carolina Department of Health and Human Services; ^2^Division of State and Local Readiness, Center for Preparedness and Response, CDC; ^3^Division of Laboratory Sciences, National Center for Environmental Health, CDC; ^4^Agency for Toxic Substances and Disease Registry; ^5^Cumberland County Department of Public Health, Fayetteville, North Carolina; ^6^Bladen County Health Department, Elizabethtown, North Carolina.

In June 2017, local health departments asked the North Carolina Department of Health and Human Services (NCDHHS) to provide health information and guidance regarding 2,3,3,3,-tetrafluoro-2-(1,1,2,2,3,3,3-heptafluoropropoxy)-propanoate (GenX) and other per- and polyfluoroalkyl substances (PFAS) that had been detected in the Cape Fear River, an important drinking water source (*1*). PFAS are a group of man-made chemicals that have been used in industry and consumer products worldwide since the 1950s. Most PFAS do not break down in the environment and can accumulate over time, resulting in increased human exposures. Limited studies in humans have indicated that some PFAS might affect reproduction, development, and the immune system and increase the risk for certain types of cancer (*2*). The source of GenX and other PFAS contamination in the Cape Fear River was a PFAS chemical manufacturing facility. After further investigation, the North Carolina Department of Environmental Quality identified GenX and other PFAS in surface water, air, and private wells close to the facility. As of April 2018, 837 private wells within a 5-mile radius of the facility had been tested; 207 (25%) had GenX levels exceeding the NCDHHS provisional drinking water health goal of 140 parts per trillion (ppt),* with a maximum measured GenX concentration of 4,000 ppt. The manufacturer began providing bottled water to residents living in homes with a well that exceeded the NCDHHS provisional drinking water health goal. In August 2018, NCDHHS worked with local health departments and asked CDC to quantify GenX and other PFAS in serum and urine specimens from a convenience sample of residents near the facility.

NCDHHS identified households near the facility with the highest concentrations of GenX in their private drinking water wells. One adult and, if available, one minor (aged 12–17 years) from each household were invited to participate. Participants had to have lived in their home full-time, used their well as their primary drinking water source before GenX detection (2017), and had no known occupational PFAS exposure. NCDHHS staff members made three call attempts to each household before contacting the next eligible household. Because the investigation was deemed to be public health epidemiologic surveillance and not research, this work was deemed exempt from institutional review board review.

Participants provided blood and spot urine specimens and completed a structured interview regarding demographics, residence history, and potential sources of PFAS exposure. CDC analyzed serum for 17 PFAS and urine for 16 PFAS. All laboratory analyses were conducted using CDC laboratory methods and established procedures for quality assurance and control (*3*). When possible, participants’ PFAS concentrations were compared with population estimates from the National Health and Nutrition Examination Surveys (NHANES) from 2015–2016 or 2013–2014 (*4*).

Among 47 contacted households, 25 (53%) were eligible and agreed to participate. Thirty residents (25 adults and five minors) participated. Participants ranged in age from 14 to 79 years (median = 52 years); half were male. All participants had lived in the county for at least 10 years and had been using bottled water for drinking for 4–14 months before specimen collection.

GenX was not detected in serum (Table) or urine of any participants. Nine PFAS were detected in serum. Median serum concentrations of perfluorohexane sulfonic acid (PFHxS, 2.1 *μ*g/L) and linear perfluorooctane sulfonic acid (n-PFOS, 5.5 *μ*g/L) were markedly higher than were those in NHANES participants (1.2 *μ*g/L and 3.2 *μ*g/L, respectively). The remaining seven PFAS were found at concentrations similar to or lower than those in NHANES specimens. Serum PFAS concentrations did not differ by sex, age, or number of years living in the county. One PFAS (perfluorohexanoic acid) was detected in one participant’s urine (0.4 *μ*g/L) close to the limit of detection (*5*); the other 15 PFAS tested in urine were not detected.

**TABLE:** Comparison of serum concentrations of per- and polyfluoroalkyl substances (PFAS) in the U.S. population with concentrations among participants (N = 30) residing near a PFAS manufacturing facility where PFAS were detected — North Carolina, 2018

PFAS	Abbreviation	Limit of detection (LOD)	Serum concentrations (*μ*g/L)				
			Participants (N = 30)			U.S. population*	
			Median	Minimum	Maximum	Median	95th percentile
2,3,3,3,-tetrafluoro-2-(1,1,2,2,3,3,3-heptafluoropropoxy)-propanoate	GenX	0.1	^—†^	^—^	^—^	Not measured	
perfluorobutane sulfonic acid	PFBS	0.1	^—^	^—^	^—^	^—^	^—^
perfluorohexanoic acid	PFHxA	0.1	^—^	^—^	^—^	Not measured	
perfluorobutanoic acid	PFBA	0.1	^—^	^—^	^—^	Not measured	
perfluoroheptanoic acid	PFHpA	0.1	^—^	^—^	0.6	^—^	0.2
perfluoropentanoic acid	PFPeA	0.1	^—^	^—^	^—^	Not measured	
4,8-dioxa-3H-perfluorononanoat	ADONA	0.1	^—^	^—^	^—^	Not measured	
9-chlorohexadecafluoro-3-oxanonane-1-sulfonate	9Cl-PF3ONS	0.1	^—^	^—^	^—^	Not measured	
2-(N-methyl-perfluorooctane sulfonamido) acetic acid	MeFOSAA	0.1	^—^	^—^	0.6	^—^	0.6
perfluorohexane sulfonic acid	PFHxS	0.1	2.1	0.7	6.7	1.2	4.9
linear perfluorooctanoic acid	n-PFOA	0.1	1.8	0.4	7.3	1.5	4.1
branched perfluorooctanoic acids	Sb-PFOA	0.1	^—^	^—^	^—^	^—^	^—^
perfluorodecanoic acid	PFDA	0.1	0.2	^—^	1.3	0.1	0.7
perfluoroundecanoic acid	PFUnDA	0.1	^—^	^—^	0.5	^—^	0.4
perfluoromethylheptane sulfonic acids (methyl branched PFOS)	Sm-PFOS	0.1	1.2	0.2	7.4	1.5	5.7
linear perfluorooctane sulfonic acid	n-PFOS	0.1	5.5	1.4	34.6	3.2	12.8
perfluorononanoic acid	PFNA	0.1	0.6	^—^	2.1	0.6	1.9

* CDC. Fourth national report on human exposure to environmental chemicals: updated tables, January 2019, volume one. Atlanta, GA: US Department of Health and Human Services, CDC; 2019. https://www.cdc.gov/exposurereport/pdf/FourthReport_UpdatedTables_Volume1_Jan2019-508.pdf.

^†^ Below the LOD (applies to all of the dashes in cells of the table).

GenX was not detected in specimens from participants with documented drinking water exposure. This might be because participants had switched to bottled water months earlier and might indicate that GenX has a relatively short half-life in humans. Compared with the general population, the higher concentrations of two historically used PFAS (PFHxS and n-PFOS) with relatively long biologic half-lives might reflect residents’ higher past or ongoing environmental exposures.

The results of this investigation provided community members with information about what was detectable in their blood and urine after learning that their drinking water was contaminated with PFAS and how to discuss their results with their primary health care provider. In addition, participants were provided general information about potential health effects from PFAS exposures. These findings might be useful to community members, public health agencies, and researchers investigating PFAS exposures and potential human health implications in the future.

## References

[1] Sun M, Arevalo E, Strynar M, Legacy and emerging perfluoroalkyl substances are important drinking water contaminants in the Cape Fear River watershed of North Carolina. Environ Sci Technol Lett 2016;3:415–9. 10.1021/acs.estlett.6b00398

[2] Agency for Toxic Substances and Disease Registry. What are PFAS? Atlanta, GA: US Department of Health and Human Services, Agency for Toxic Substances and Disease Registry; 2018. https://www.atsdr.cdc.gov/pfas/overview.html

[3] Kato K, Kalathil AA, Patel AM, Ye X, Calafat AM. Per- and polyfluoroalkyl substances and fluorinated alternatives in urine and serum by on-line solid phase extraction-liquid chromatography-tandem mass spectrometry. Chemosphere 2018;209:338–45. 10.1016/j.chemosphere.2018.06.085 29935462 PMC7916321

[4] CDC. Fourth national report on human exposure to environmental chemicals: updated tables, January 2019, volume one. Atlanta, GA: US Department of Health and Human Services, CDC; 2019. https://www.cdc.gov/exposurereport/pdf/FourthReport_UpdatedTables_Volume1_Jan2019-508.pdf

[5] North Carolina Department of Health and Human Services. Biological sampling for GenX and other per- and polyfluoroalkyl substances (PFAS)—North Carolina, 2018. Raleigh, NC: North Carolina Department of Health and Human Services; 2018. https://epi.dph.ncdhhs.gov/oee/pfas/NCDHHS_PFAS%20Biomonitoring%20Report_8Nov2018.pdf

